# “Skin Popping” and “Shooter’s Patch” As Manifestations of Intradermal Drug Abuse

**DOI:** 10.7759/cureus.45251

**Published:** 2023-09-14

**Authors:** Archana Samynathan, Kaarl Saardi, Yagiz M Akiska, Alana Sadur, Skylar Johnson, Mana Nasseri

**Affiliations:** 1 Department of Dermatology, George Washington University School of Medicine and Health Sciences, Washington, D.C., USA; 2 Department of Dermatology, Bangalore Medical College and Research Institute, Bangalore, IND

**Keywords:** injectable drug abuse, physician education, systemic complications, foreign body granulomas, talc

## Abstract

Talc, a common adulterant in injectable opioids and filler in oral tablets, is frequently abused as crushed suspensions in injections. This review aims to recognize intradermal drug injection referred to colloquially as “skin popping” or “shooter’s patch” as a cause of granulomatous disease and prevention of systemic complications from cutaneous cues.

## Introduction and background

The use of illicit drugs has been a persistent and longstanding addictive public health issue. The number of persons who inject drugs (PWID) has grown sharply in recent years [1]; the estimated number of PWID in the United States was 3 694 500 in 2018 [2]. The global estimate of PWID was 13.2 million and overall illicit drug use was estimated at 296 million in 2021, an increase of 23% over the previous decade. The number of people who suffer from drug use disorders has skyrocketed to 39.5 million, a 45% increase over 10 years [3].

Injecting drug use (IDU) is responsible for many life-threatening and fatal complications and the skin is the organ most evidently affected. Talc is an industrial mineral whose chemical composition is silicon dioxide (SiO_2_) (63.5%), magnesium oxide (MgO) (31.7%), and water (H_2_O) (4.8%) [4]. Heroin is adulterated with many compounds that resemble the pure powder, both physically and in solubilizing properties before it reaches the users [5]. This is necessary because heroin is administered either intravenously, subcutaneously, or intradermally, which is termed “skin popping” by heroin users. Talc also acts as a filler and lubricant in tablets containing oral medications [5]. Oral drug tablets like amphetamines, methylphenidate, propoxyphene, methadone, morphine, and benzodiazepines where the principal filler is talc are crushed, melted, dissolved in water, and intravenously injected. Suspended microscopic talc extravasates into the skin leading to foreign body granuloma in the skin. Microscopic talc particles may also lodge in the vessels of various organs with rich blood supply, migrate to the interstitium, and lead to systemic foreign body granulomatosis (FBG) due to talc’s insoluble nature and fibrogenic properties [6,7].

It is well established that large particles of silica do not produce granuloma but the smaller particles, less than 100 nm in the colloidal form, produce granuloma in all persons. The time between the introduction of the foreign body into the skin and the granuloma formation is variable [7]. Typically, the period is long and the variability is explained by the conversion of silica (i.e., sand) or silicates (i.e., talc) to colloidal form that may take many years, thus further emphasizing the need for meticulous history investigating remote drug use and physical exam. There are reports of skin nodule appearances 20-30 years after cessation of intravenous drug use [5,8,9].

## Review

Clinical manifestations

Patients with FBG due to talc have clinical presentations ranging from asymptomatic to fulminant disease including lupus erythematosus (LE), vasculitis, sarcoidosis, and dermatomyositis, presenting with nonspecific complaints, including progressive exertional dyspnea, dry or productive cough, fatigue, and visual disturbances [4]. However, due to the high incidence of misplaced injections (mostly transdermal) self-administered by PWID, cutaneous manifestations of FBG have been increasing in number [10].

**Figure 1:**
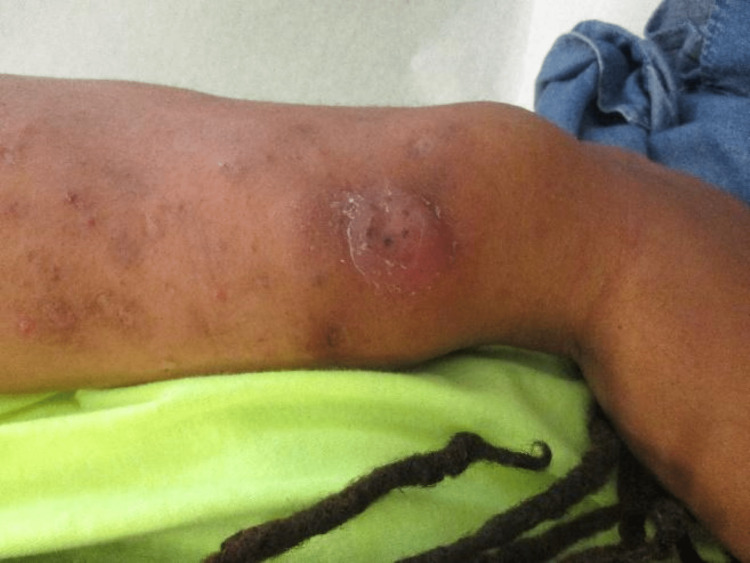
Cutaneous Granuloma Image Credit: Authors

**Figure 2:**
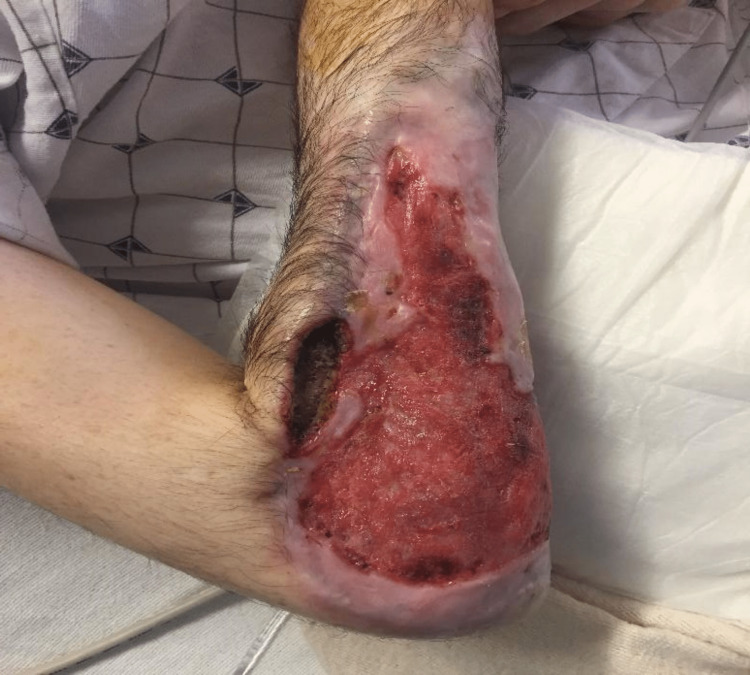
Extensive Ulceration Image Credit: Authors

Drugs adulterated with talc used by PWID have been associated with the formation of granulomas in the skin, commonly presenting as either skin-colored or discolored (pink, red-brown, purple depending on the skin type) papules, nodules, or plaques followed by papulovesicular lesions (Figure 1). Shooter’s patch or skin popping is a tell-tale sign of intradermal or subcutaneous present or past drug use. Present use resembles needle pricks, erosions, ulcers, or wounds, past use mostly present as irregular circular depressed scars or keloids with dyspigmentation. Skin lesions can be tender or asymptomatic in nature. They may be accompanied by abscesses, overlying ulcers, and rarely regional lymphadenopathy (Figure 2). Granulomas in the skin can develop months or years after the last injection [11]. A detailed skin exam can reveal other cutaneous signs of drug abuse such as carbon tattoos (“track marks”) with or without scarring, subcutaneous abscesses, and thrombophlebitis.

The clinical presentation of FBG due to talc may depend on tissue response, anatomical site, penetration, composition, and amount of material involved [12]. Immune-compromised PWID, such as those with HIV infection, are also at risk for secondary infections. Distinguishing infections that cause granulomatous inflammation (such as tuberculosis, non-tuberculous mycobacteria, fungi, and fastidious bacteria) can be extremely challenging. 

While cutaneous lesions may not seem disabling, talc granulomatosis can cause life-threatening cardiopulmonary and neurologic complications. Presenting symptoms can include dyspnea and cough. Symptoms can be the result of pulmonary nodules, fibrosis, emphysema, or pulmonary arterial hypertension (PAH). PAH is caused by microparticles less than 10 micrometers passing through the pulmonary vasculature and leading to right-sided heart failure.

Talc is also a rare known cause of liver granulomas where birefringent microcrystals can be detected in hypertrophied portal macrophages [13]. Finer talc crystals have also been described in the retina, cardiac tissue, mediastinal lymph nodes, spleen, muscle, kidney, skin, pancreas, and bone marrow [14]. It is critical to specifically check for IDU, potential immunocompromised conditions, occupational exposure, and accidents during history-taking since these are high-risk factors for talc exposure [7].

Diagnosis and differential diagnosis

**Figure 3:**
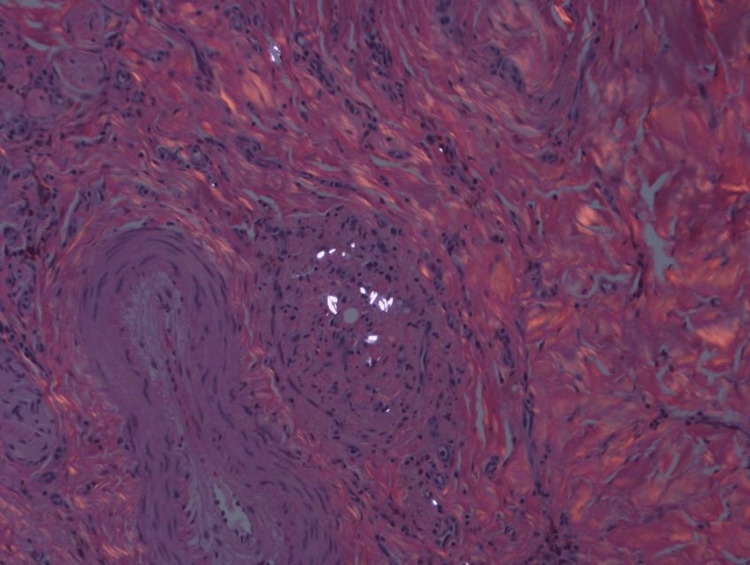
Hematoxylin & eosin stain under polarizing lens visualizing the highly birefringent talc granuloma Image Credit: Authors

Biopsy of affected tissue is the gold standard to confirm the diagnosis. Imaging studies are not recommended due to the small size of cutaneous foreign bodies. Granulomas are organized collections of macrophages that form a compact structure in response to infections, inflammatory stimuli, and foreign bodies [15], comprising epithelioid histiocytes and multinucleated giant cells on histology [3]. If there is enough talc material present in the granuloma, it can be visualized via hematoxylin & eosin stain; however, a polarizing lens can be used for better visualization due to the high birefringence (Figure 3) of talc’s sheet/plate-like material [16]. In differentiating between talc and microcrystalline cellulose, another common adulterant and filler or binding agent, a modified Russell Movat Pentachrome stain may be used [17]. This stains talc light blue and microcrystalline cellulose yellow. Skin findings can be divided into four histopathological groups: leukocytoclastic vasculitis (LCV), dermal pigment deposition, necrobiosis lipoidica-like dermatitis, and non-specific ulceration/scarring [18]. It is important to rule out other granulomatous conditions including infections, and sarcoidosis, using tissue cultures [9].

Treatment

Currently, there are no established treatments for talc granulomatosis besides supportive care, topical corticosteroids, and the use of antibiotics when deemed necessary [19,20]. Further, work-up for pulmonary and cardiac involvement must be prioritized as they may be asymptomatic initially and yet can have the most life-threatening consequences. There are reports of granuloma being found on autopsies. Thus, early detection is critical to prevent a progressive poor prognosis. X-rays, high-resolution CT (HRCT), abdominal CT, and urinalysis microscopy fundoscopy where microcrystals pass through and are excreted are some of the initial significant work-up tests. Frequent dermatological and/or pulmonary follow-up is highly recommended to monitor and prevent potential systemic events. The chief component of treatment is to correctly identify IDU status as part of a psychiatric illness and refer the patient for psychological counseling for IDU cessation [11]. Cessation of newer lesions on abstinence has been reported.

## Conclusions

Clinical presentation varies depending on the patient. Dermatologists need to be aware of all the cutaneous manifestations of drug abuse because of the benefits of early diagnosis and therapeutic intervention. Cutaneous granulomas due to IDU are a serious medical problem as they can provoke suspicion of subclinical systemic illness. Cutaneous granulomatosis is frequently difficult to diagnose due to its heterogeneous etiologies in clinical practice. This makes patient history and physical examination findings extremely critical in guiding potential talc-exposure diagnosis. It is important to note that due to a potential delayed onset, it may be challenging for patients to recall exposure.

It is crucial to associate IDU, potential immunocompromised conditions, occupational exposure, and accidents in the history since these are high-risk factors for talc exposure. Treatment for cutaneous granuloma should be approached on a case-by-case basis. A skin biopsy may be done at the first visit, to diagnose early on as follow-up is an issue with addictive behavior. When foreign bodies suggestive of talc are seen, patients may be sent for further imaging including chest C-ray and/or HRCT, abdominal CT, and urinalysis and microscopy as talc granulomas are likely to manifest in the cardiopulmonary tissue as a potentially common life-threatening systemic disease, especially in the lungs. Good quality care will include attempts to assess systemic involvement and most importantly offer deaddiction.
